# The aggregation, rheological and structural properties of casein-dextran colloids induced by critical-zone-intensity ultrasound^☆^

**DOI:** 10.1016/j.ultsonch.2025.107385

**Published:** 2025-05-23

**Authors:** Binsha Peng, Songlin Wen, Wenchong He, Chongde Wu, Jun Huang, Rongqing Zhou, Nicolas Hengl, Frederic Pignon, Yao Jin

**Affiliations:** a College of Biomass Science and Engineering, Sichuan University, Chengdu 610065, China; b Key Laboratory for Leather and Engineering of the Education Ministry, Sichuan University, Chengdu 610065, China; c Research Management Office, West China Second University Hospital, Sichuan University, Chengdu, China; d National Engineering Research Center of Solid-State Manufacturing, Luzhou 646000, China; e Univ. Grenoble Alpes, CNRS, Grenoble INP (Institute of Engineering Univ. Grenoble Alpes), LRP, F-38000 Grenoble, France

**Keywords:** Casein-dextran colloids, Ultrasound, Aggregation properties, Rheological properties, Structural properties

## Abstract

Casein-dextran colloids, with various particle and filtration properties to simulate the main pollutant (protein-polysaccharide colloids) in the membrane separation process, were selected as the research object in this study. Effects of critical-zone-intensity ultrasound (CZ-US) on the aggregation, rheological and structural properties of casein-dextran colloids were investigated by ultrasonic immersion. The results suggested that CZ-US treatments unfolded the protein tertiary/quaternary structure to expose hydrophobic/hydrophilic amino acid groups, and partially affected the aggregation and flow behaviors, without altering the secondary structures of proteins and functional groups for casein-dextran colloids. CZ-US treatments enabled particle size distribution of casein-dextran colloids transform from multimodal to unimodal. CZ-US treatments’ processes were strongly affected by pH of the colloidal mediums. When pH was at the casein isoelectric point, more hydrophobic amino acid groups of casein were exposed, the binding patches on the casein particle interface were reduced for the dextran. When pH was at other mediums rather than the casein isoelectric point, more hydrophilic amino acid groups were exposed under CZ-US treatments, more dextran adhered into the casein interface due to the presence of more available binding sites. Indeed, this work provides important theoretical guidance for the development of CZ-US and membrane separation coupling technology.

## Highlights

• Critical-zone-intensity ultrasound (CZ-US) unfolded the protein.
• No alteration of secondary structures and functional groups under CZ-US.
• CZ-US mildly affected the aggregation and flow behaviors for casein-dextran colloids.
• More hydrophilic groups were exposed under CZ-US at casein PI.
• More hydrophobic groups were exposed under CZ-US at casein ≠ PI.

## 1 Introduction

The protein-polysaccharide colloids, composed of natural origins and classified as macromolecular biopolymers [1], bind with different interaction to form networks [2,3]. Moreover, inherent characteristics of protein and polysaccharides, such as particle properties [1] or rheological properties [4], determined properties of protein-polysaccharide colloids. As an important representative system, protein-polysaccharide colloids are involved in broad research and applications. Special protein-polysaccharide colloids were developed as emulsifiers [5] or composite materials [6] in food industry [7], pharmaceutical industry [8], cosmetics industry [9], and they have also been identified as the main foulant in membrane separation process due to their complex processing behavior [10].

In recent years, the coupling technology of ultrasound and membrane separation is an important research direction to control membrane fouling [11]. Pétrier et al [12] classified ultrasound: the ultrasound of less than 1 W in output power is defined as low-intensity ultrasound, the ultrasound of greater than 10 W in output power is defined as high-intensity ultrasound (HI-US), then the output power between 1 W and 10 W is categorized into critical-zone-intensity ultrasound (CZ-US). Relevant studies [13,14] have pointed out that HI-US membrane separation coupling could improve the filtration efficiency by enhancing the mass transfer. However, numerous studies [15,16] have reported that HI-US treatment affected protein-polysaccharide colloid properties (particle, rheological, and structural properties). Namely, HI-US may alter the inherent properties of the feed colloids, then properties and processing behaviors of protein-polysaccharide colloids could be influenced by several extreme effects due to HI-US treatments. For instance, HI-US can bring about continuous heat effect (converted from the internal friction between the medium) and instantaneous heat effect (generated by the acoustic cavitation) [17], which enable protein structure unfold [18]. Albano and Nicoletti [15] reported that ultrasound of 320 W reduced suspension viscosity and the size of whey protein-pectin complexes. Furthermore, HI-US can increase the amount of surface charge of whey protein concentrate and pectin, so that the electrostatic interaction among protein and polysaccharide complexes could be strongly modified [19]. In a more recent work, the α-helix fraction in protein decreased significantly upon HI-US treatment, accompanied by a paralleled increase in β-sheet and unordered [20].

Considering the above disadvantages, our previous studies [16] revealed that the applied CZ-US (20 kHz, 2 W–10 W) led to a significant increase of permeate flux arising for casein micelles from a disruption of concentrated layer. Chávez-Martínez et al. [21] had showed poor damages of 3 W/cm^2^ ultrasound on dairy product’s properties, which consolidated the application potential of coupling CZ-US with membrane separation in treating protein-polysaccharide colloids. Nonetheless, there is little know about the effects of CZ-US on the inherent properties of protein-polysaccharide colloids.

Previously, our group [10] had investigated casein-dextran colloids under different solution conditions to simulate the primary pollutant that cause membrane fouling in different processing industries. From colloids with different pH (2.8, 4.8, 6.8, and 7.8) and casein/dextran content ratio (casein: dextran = 4:1, 1:1, 1:4, 1:9), we have highlighted four representative casein-dextran colloids of distinctive characteristics: Colloid I (multimodal particle distribution, pH 2.8, casein: dextran = 1:1) and Colloid II (unimodal particle distribution, pH 4.8, casein: dextran = 1:4: showing certain yield stress with low total concentration of protein and polysaccharide; Colloid III (unimodal particle distribution, pH 4.8, casein: dextran = 4:1): shear thinning behavior, showing the highest total fouling resistance and fouling growth rate; Colloid IV (multimodal particle distribution, pH 7.8, casein: dextran = 1:1): Newtonian fluid, showing low total fouling resistance and the smallest pollution growth rate.

Therefore, those four casein-dextran colloids were selected as the research object in this study. Effects of CZ-US on the aggregation, rheological and structures properties of those casein-dextran colloids were investigated. Indeed, this work provided the theoretical guidance for the new direction of CZ-US −membrane separation coupling, as well as an important reference for the response of colloids in this specific ultrasonic zone field.

## 2 Materials and methods

### 2.1 Materials

Protein-polysaccharide colloids used in the experiments were casein (protein isoelectric point (pl) = 4.8) powder (high purity grade, > 95 %) from bovine milk (Cool Chemistry, HJ531622) and dextran (Cool Chemistry, L2000704, MW≈15 kDa).

### 2.2 Sample preparation

This study combined our previous research [10] to determine the experimental variables for this experiment. The stock solution of casein (10 g/L) was prepared by mixing the casein powder from bovine milk in a 0.04 M sodium hydroxide solution, which was stirred (250 r/min) for 4 h. A stock solution of dextran (10 g/L) was prepared by mixing the dextran powder in DI water. Table 1 shows the specific value of each variable for the tested colloids. Buffer solutions were prepared with various pH, IS (strength of ions, represented by the concentration of NaCl) and Ca^2+^ (CaCl_2_) concentrations. Meanwhile casein and dextran mixing solutions were stirred (250 r/min) for 10 min, then poured it into a buffer solution under specific conditions and stirred (250 r/min) for 10 min. The same hybrid approach has been used in precedent work [22].

**Table 1:** Solution conditions of experimental samples (casein-dextran colloids).

Casein-dextran colloids	Rigid conditions	Variable conditions
Colloid Ⅰ	IS = 0.10 M; c (Ca^2+^) = 1.0 mM	pH 2.8; casein: dextran = 1:1
Colloid Ⅱ		pH 4.8; casein: dextran = 1:4
Colloid Ⅲ		pH 4.8; casein: dextran = 4:1
Colloid Ⅳ		pH 7.8; casein: dextran = 1:1

Note: IS = c(NaCl); casein: dextran = c: c; The total concentrations of casein-dextran colloids were 1 g/L.

### 2.3 CZ-US treatment

**Fig. 1:**
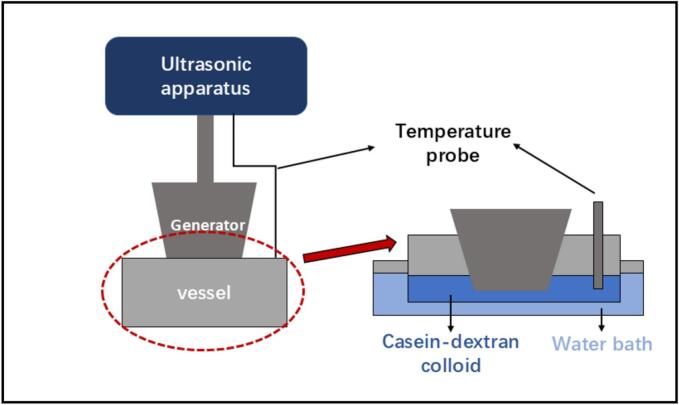
Schematic overview of CZ-US set-up.

Fig. 1 indicates a schematic diagram of the CZ-US set up. Casein-dextran colloids were put in a square vessel to be processed with a ultrasonic probe (Scientz-35D, NINGBO SCIENTZ BIOTECHNOLOGY Co., LTD, China), and the ultrasonic apparatus takes a maximum output power of 10 W and an operating frequency of 35 kHz. The temperatures of reactants were maintained with a water bath at 25 ± 2 ℃. Samples were subjected to ultrasound for 60 min at 1 W and 10 W with 1 s/1 s (1 s on 1 s off on pulse mode), corresponding to power intensity at 0.25W cm^−2^ and 2.5 W cm^−2^ (the input power per unit area of the blade surface).

### 2.4 Properties of casein-dextran colloids

#### 2.4.1 Protein solubility measurement

Protein solubility was determined by using the Coomassie brilliant blue method. The protein absorbance in the reactor was determined at 595 nm, using a spectrophotometer (L7, INESA, China). Bovine serum albumin (BSA) was used as the standard. Protein solubility was calculated as the percentage of soluble protein in the colloid relative to the total protein content in the sample.

#### 2.4.2 Rheometric, Size, zeta potential and turbidity measurements

Rheological behaviors of casein-dextran colloids were measured with modular compact rheometer (Anton Paar, MCR 302) at 25℃. For the colloids with the rotor of cone plate geometry, equipped with CP50-1 cone (diameter 50 mm, angle 1°). The shear rate was raised from 0.1 to 300 s^−1^ over the period of measurement. Zeta potential and particle size were measured with Malvern Mastersizer (ZEN3600 + MTP2, Malvern, UK). This system measures electrophoretic mobility of the particles and automatically calculates zeta potential by the Henry equation. The turbidity of samples was measured with portable turbidity meter (WZS-186, INESA, China) in the nephelometric turbidity units (NTU).

#### 2.4.3 Ultraviolet–visible (UV–vis) spectroscopy

The ultraviolet–visible spectra of casein-dextran colloids, with and without ultrasound treatments, were recorded in the wavelength range of 200–500 nm, using an ultraviolet visible light spectrophotometer (L7, INESA, China) at room temperature (25 ± 1 °C) with a 1.0 cm path length quartz cuvette, 100 nm/min scan rate and 1.0 nm bandwidth. The spectrum of deionized water was used as blank.

#### 2.4.4 Circular dichroism (CD) spectroscopy

Circular dichroism (CD) spectra of casein-dextran colloids, with and without ultrasound treatments, were measured by a CD spectropolarimeter (J-500C, Japanese spectroscopic industry, Japan). Using a quartz cuvette of 1 mm optical path length at 20 ± 2 °C, CD spectra were scanned at a wavelength of the far UV range (260–190 nm), with three replicates, at speed of 50 nm/min and interval of 1 nm. The secondary structure elements (α-helix, β-sheet, β-turn, random coil) of samples were analyzed using DICHROWEB.

#### 2.4.5 Fourier transform infrared spectroscopy

The samples were frozen and set at −80 ℃, and then frozen and dried in a freeze-dryer (LGJ-1D, YATAIKELONG, China) for 3–––4 days until the samples became powders. Functional groups characteristics of casein-dextran colloids were measured through the Fourier transform infrared spectrometer (FTIR) by the method of powder tableting, and spectra results were obtained in the 4000 ∼ 400 cm^−1^ region (IRTracer-100, The Japanese island ferry, Japan).

#### 2.4.6 Confocal laser scanning microscopy (CLSM)

A laser scanning confocal microscope (LSM880, Zeiss, Germany) was used to observe the microstructure of the casein-dextran colloids. Firstly, colloids were incubated with the casein stain 10 mg/L fluorescein isothiocyanate (FITC, S25030, yuanye Bio-Technology, China) and dextran stain 5 mg/L concanavalin A-AF555 （Con A-AF555, 2551203, AAT Bioquest, USA）for 30 min at 37 ℃ in dark. Then, the incubated colloids were immediately observed by CLSM with an amplification of 400 times. Meanwhile, FITC was excited by a 488 nm laser with a receiving light range from 493 nm to 560 nm. Con A-AF555 was excited by A 561 nm laser with A receiving light range from 566 nm to 697 nm.

### 2.5 Statistical analysis

Triplicate in experiments were conducted on each sample to ensure good repeatability and the data was represented in the form of means ± standard deviation (SD). Statistical Program of Social Science (SPSS 17.0, Chicago, IL, USA) software was used for multi-factor analysis of variance to explore the influence degree of each factor, at a significant level of P < 0.05. Meanwhile, the partial Eta square expresses the effect size. Three different batches (n = 3) were considered and analyzed throughout the study.

## 3 Results

### 3.1 The aggregation of casein-dextran colloids induced by CZ-US

#### 3.1.1 Change in the solubility of protein

Solubility is the most practical measure of protein aggregation [23]and affects many important functional properties of protein-polysaccharide colloids.

**Fig. 2:**
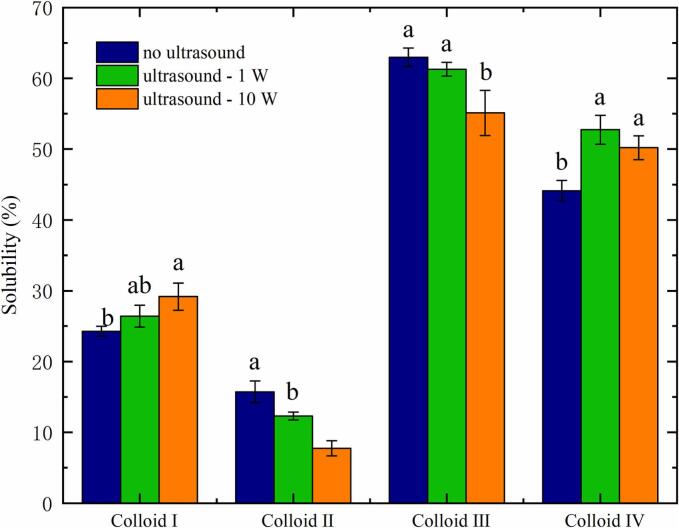
The effect of different ultrasonic powers on the protein solubility of colloids. Different letters indicate that they are significantly different at p < 0.05. ANOVA analysis was applied.

Fig. 2 reveals that the solubility of the casein at pH 2.8 (Colloid Ⅰ) was lower than pH 7.8 (Colloid Ⅳ), which may result from the exposure of hydrophobic groups after the casein structure unfolds under acidic treatment [24]: namely to exposed more hydrophobic groups in Colloid Ⅰ (pH 2.8) than Colloid Ⅳ (pH 7.8). Meanwhile, the solubility of casein for all colloids were different, and it was obviously impacted by 10 W CZ-US treatments. When the applied ultrasonic power was increased from 1 to 10 W, the solubility of casein in Colloid Ⅱ and Ⅲ (pH at the casein isoelectric point) showed a tendency to decrease, while the solubility of casein in Colloid Ⅰ (pH 2.8) and Ⅳ (pH 7.8) increased with CZ-US treatments under pH outside the isoelectric point. High intensity ultrasound leads to inertial cavitation with microbubble collapse, while low intensity ultrasound leads to stable cavitation of microbubbles [25]. Meanwhile, lots of cavitation bubbles during ultrasound treatment brings high temperature and pressure in local, which makes protein structure unfold leading to exposure of hydrophobic/hydrophilic groups [23]. Therefore, change in the solubility of protein induced by CZ-US treatment may be related to the exposure of hydrophobic/hydrophilic amino acid groups of proteins.

#### 3.1.2 Particle size distribution

Notably, the changing protein solubility may lead particle size distributions of protein-polysaccharide colloids to change. Thus, in this section, size distributions of casein-dextran colloids’ particle were investigated under CZ-US treatments with different powers.

**Fig. 3:**
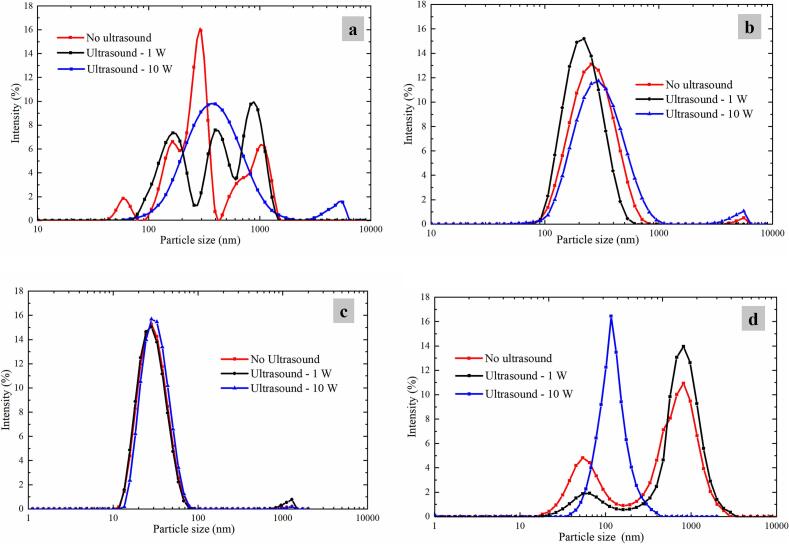
Particle size distributions of colloids (Colloid Ⅰ (a), Colloid Ⅱ (b), Colloid Ⅲ (c) and Colloid Ⅳ (d)) treated by different ultrasonic powers.

Fig. 3 shows that particle size distributions of Colloid Ⅰ (pH 2.8) and Colloid Ⅳ (pH 7.8) were multimodal compared with Colloid Ⅱ and Ⅲ (pH 4.8, at pl) dealing with no ultrasound. Meanwhile, there is an obvious transformation of particle size distribution from multimodal to unimodal increasing ultrasonic powers for Colloid Ⅰ (pH 2.8, shown in Fig. 3a) and Colloid Ⅳ (pH 7.8, shown in Fig. 3d). Ultrasonic homogenization occurs through two main mechanisms: generation of an acoustic field and acoustic cavitation phenomenon [26,27]. The acoustic field generates unstable interfacial waves resulting in dispersed colloidal particles in the liquid phase [28]. The stable cavitation phenomenon induced by CZ-US produces localized high temperatures that promote the aggregation of smaller polymer particles [29], as well as localized pressure that induces the fragmentation of larger polymer particles [30]. However, the CZ-US treatment of this work did not reveal a consistent trend in the variation of colloidal mean particle sizes, likely attributable to the counterbalancing effects between particle dispersion and aggregation phenomena induced by CZ-US.

#### 3.1.3 Zeta potential and turbidity

**Fig. 4:**
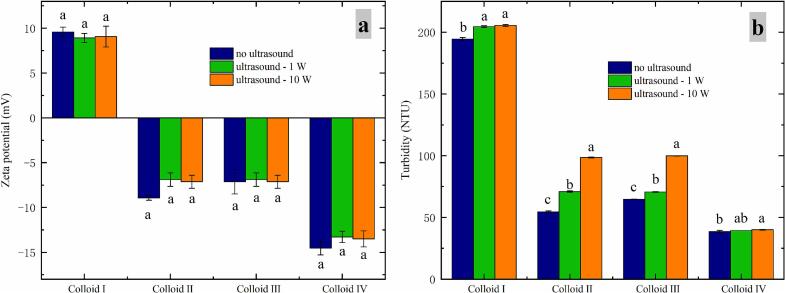
Effects of different ultrasonic powers on the zeta potential (a) and turbidity (b) of colloids. Different letters indicate that they are significantly different at p < 0.05. ANOVA analysis was applied.

The particle size is the most important factor to affect turbidity [31], and is also affected by surface charge of the system [32]. Therefore, zeta potential and turbidity of casein-dextran colloids under the application of CZ-US were discussed in this section. CZ-US treatments had no significant influence on surface charge of casein-dextran colloids (Fig. 4a). Beyond that, the order of zeta potential absolute value (Z) for colloids were: Colloid Ⅲ < Colloid Ⅱ < Colloid Ⅰ < Colloid Ⅳ. A low Z [33] of one colloidal system indicate its relatively poor stability. Therefore, the order of stability for colloids were: Colloid Ⅲ < Colloid Ⅱ < Colloid Ⅰ < Colloid Ⅳ. Concerning the turbidimetric analyses, turbidity of casein-dextran colloids increased under CZ-US with increasing ultrasonic powers (Fig. 4b), and the highest variation occurred for Colloid Ⅱ and Ⅲ with the weakest stability. On the opposite, the lowest variation occurred for Colloid Ⅳ with the strongest stability. Namely, the more unstable the system is, the greater the influence of CZ-US on its turbidity.

### 3.2 The rheological properties of casein-dextran colloids induced by CZ-US

**Fig. 5:**
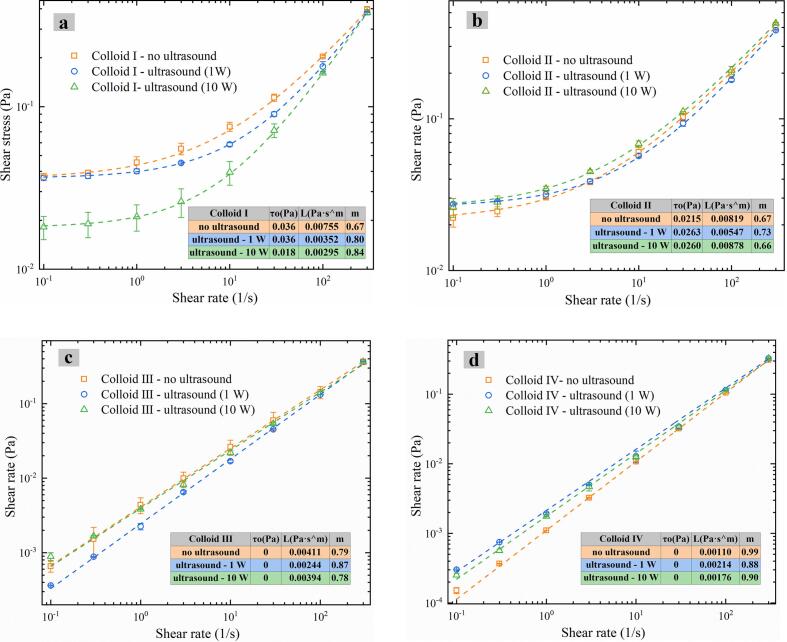
Rheological curve of colloids (Colloid Ⅰ (a), Colloid Ⅱ (b), Colloid Ⅲ (c) and Colloid Ⅳ (d)) treated by different ultrasonic powers.

Ultrasound treatments can change the degree of aggregation for protein and polysaccharide molecules in the solution, which in turn affects the rheological properties of protein-polysaccharide colloids (Šegota et al., 2006). Thus, relationships between the shear stress and shear rate of casein-dextran colloids with CZ-US process are presented in Fig. 5, illustrating evolutions of rheological behavior for such ultrasound-treated colloids.

Specially, Colloid Ⅰ (pH 2.8, casein: dextran = 1:1, Fig. 5a) and Colloid Ⅱ (pH 4.8, casein: dextran = 1:4, Fig. 5b) exhibited gel consistency and yield stresses on the flow curve fitted by a Herschel-Bulkley viscoelastic model:

(1) $$\tau =\tau_{0}+L{\dot{\gamma }}^{m}$$

where τ represents the shear stress (Pa), L is the consistency coefficient (Pa s^m^), $\dot{\gamma }$ is the shear rate (s^−1^), m is the shear-thinning index and τ_0_ is the yield stress (Pa). As for Colloid Ⅰ (pH 2.8, casein: dextran = 1:1), when the pH of the solution is lower than the pl (4.8) of casein, casein can achieve maximum aggregation yield [34], forming a gel network. Otherwise, the addition of high concentrations of polysaccharide may increase the viscosity of the colloid and rearrange the protein structure, making it easy for gelation [35], so Colloid Ⅱ (pH 4.8, casein: dextran = 1:4) also exhibited yield stresses.

In addition, Fig. 5c and Fig. 5d show that shear-thinning behaviors (m < 1) or Newtonian (m = 1) were well depicted by a power law:

(2) $$\tau =L{\dot{\gamma }}^{m}$$

Effects of CZ-US treatment on the flow behavior of different casein-dextran colloids were small and specific (Fig. 5). For pH at casein isoelectric point, τ_0_ of Colloid Ⅱ (casein: dextran = 1:4) increased with CZ-US treatments (shown in Fig. 5b), and the L firstly decreased and then recovered induced by rising CZ-US power for Colloid Ⅲ (casein: dextran = 4:1, Pseudoplastic fluid, shown in Fig. 5c). For other pH mediums, τ_0_ of Colloid Ⅰ (pH 2.8, casein: dextran = 1:1, Yield stress fluid) increased slightly and then decreased with rising CZ-US power (shown in Fig. 5a). The rheological property of Colloid Ⅳ (pH 7.8, casein: dextran = 1:1) was evolved from Newtonian fluid (m ≈ 1) to pseudoplastic characteristic (shear thinning behavior, m < 1), shown in Fig. 5d. Extensive research[36,37] has demonstrated that ultrasound irradiation can significantly alter the rheological properties of polymers through modifications in their aggregation states and network structures. Consequently, the distinct rheological responses observed in the four colloidal systems under CZ-US treatment may be attributed to the exposure and conformational changes of hydrophilic and hydrophobic amino acid residues, as will be comprehensively discussed in subsequent sections.

### 3.3 The structure of casein-dextran colloids induced by CZ-US

In the above, the macroscopic properties (particle and rheological properties) of casein-dextran colloids were analyzed under CZ-US treatments. In this section, the microstructure properties were further discussed as follow.

#### 3.3.1 Microstructures of four casein-dextran colloids

**Fig. 6:**
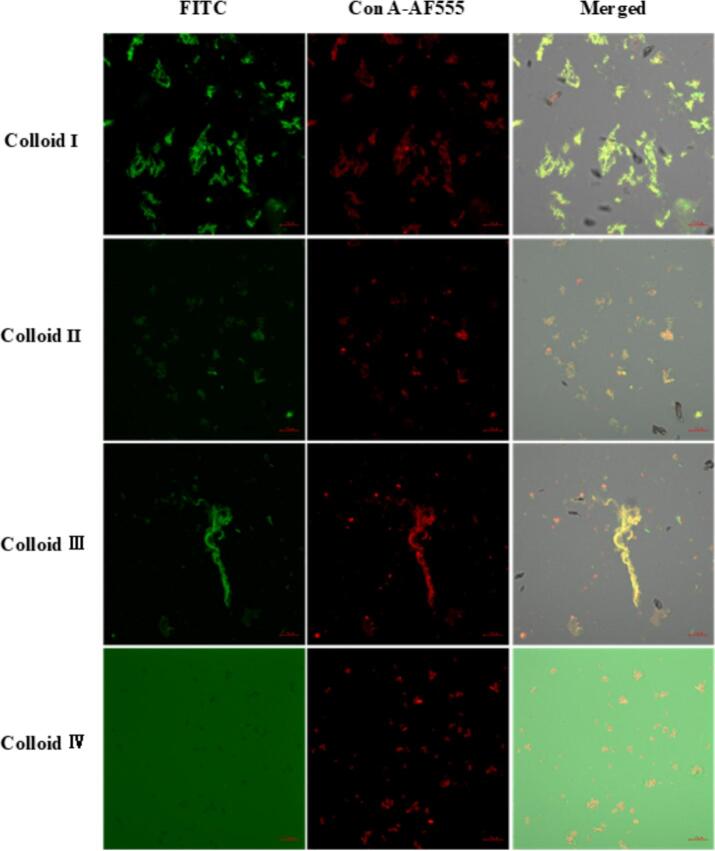
CLSM images of various casein-dextran colloids. (Casein was stained green with FTIC and dextran was stained red with ConA-AF555, and then the two images were superimposed to obtain the merged image).

Microstructures of casein-dextran colloids in various solution conditions were observed by CLSM on 20 μm length scale (shown in Fig. 6). The micrograph indicated aggregation and flocculation of the colloids. The casein was stained green by FITC, and the dextran was stained red by Con A-AF555. The mixing of casein and dextran showed different color with various casein/dextran ratios.

As shown in Fig. 6, four colloids in various solution conditions (pH mediums and casein/dextran ratios) showed different microstructure. The pH-dependent transition between soluble and insoluble aggregates was primarily governed by electrostatic effects [24]. This phenomenon can be attributed to protein charge alterations, which are associated with structural unfolding and subsequent exposure of hydrophobic/hydrophilic groups from the protein interior [38]. Specifically, proteins exhibit distinct surface structures and varying degrees of hydrophobic/hydrophilic group exposure depending on the pH environment relative to their pl. At pH values corresponding to the pl, protein polarity is minimized, facilitating the aggregation of non-polar hydrophobic groups [39,40]. For Colloid Ⅰ (discussed in section 3.1.1) and Ⅱ, casein demonstrated low solubility (Fig. 2) facilitating its aggregation through hydrophobic interactions. When casein/dextran ratio was 1:1, parts of dextran molecules in Colloid Ⅰ (pH 2.8) appeared to penetrate and adhere to the extensive casein network, forming a large composite network structure (light green fluorescence). In contrast, casein in Colloid IV (casein: dextran = 1:1) maintained high solubility (Fig. 2), remaining dispersed in aqueous medium without forming complexes with dextran aggregates at pH 7.8. In Colloid II (pH 4.8, at pl, casein: dextran = 1:4), abundant dextran molecules seemed to adhere into the small casein network to form many small uniform-sized network structures (orange fluorescence). Colloid III (pH 4.8 at pl, casein: dextran = 4:1) exhibited limited dextran crosslinking, with molecules adhering to the surface of dense, self-aggregated casein structures (yellow fluorescence).

#### 3.3.2 Ultraviolet–visible spectroscopy

**Fig. 7:**
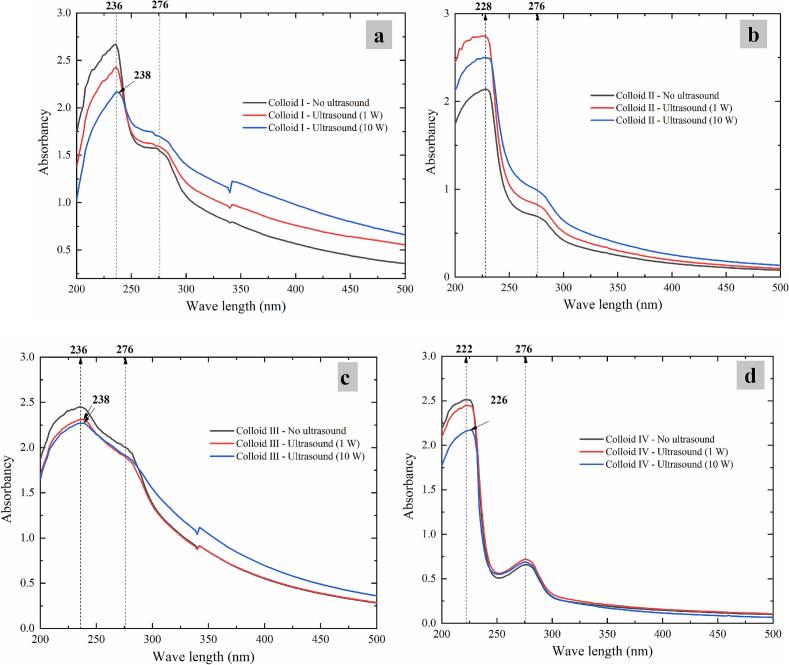
UV–vis spectroscopy of colloids (Colloid Ⅰ (a), Colloid Ⅱ (b), Colloid Ⅲ (c) and Colloid Ⅳ (d)) treated by different ultrasonic powers.

Structural changes in protein-polysaccharide colloids can be analyzed by determining the positions of ultraviolet absorption peaks at the near-ultraviolet region from 200 nm to 500 nm (containing most absorption ranges of aromatic amino acids) [41]. Fig. 7 shows the ultraviolet–visible spectroscopy of casein-dextran colloids with and without CZ-US applications. A strong absorption peak observed in the region of 220–240 nm (Fig. 7) could explain the characteristics of carbonyl group and peptide bond [42]. The characteristics broad peak (Fig. 7d) and broad shoulder peak (Fig. 7a, b and c) around 276 nm was attributed to the absorption of tyrosine, tryptophan and phenylalanineSimonian and H., [43]. Under CZ-US applications, the absorbance intensity of Colloid Ⅰ, Ⅱ and Ⅳ characteristics peak around 276 nm showed a slight increase (Fig. 7a, b and d), which illustrated that more buried tyrosine (hydrophilic), tryptophan and phenylalanine (hydrophobic) of these colloids were exposed induced by CZ-US treatments. Following CZ-US treatment, the characteristic peaks of ultrasonic Colloids I, III, and IV exhibited significant red shifts (P < 0.05), with the absorption maxima shifting from 236 nm to 238 nm (Fig. 7a), 236 nm to 238 nm (Fig. 7c), and 222 nm to 226 nm (Fig. 7d), respectively. To sum up, the CZ-US treatments slightly changed the molecular structure of these colloids [42]. The results of this work give further support to the idea that CZ-US treatment slightly changes the structure of protein-polysaccharide colloids.

#### 3.3.3 CD analysis

The far-UV CD spectra of different casein-dextran colloids were measured to analysis variations in the secondary structure of casein with different CZ-US treatments.

**Fig. 8:**
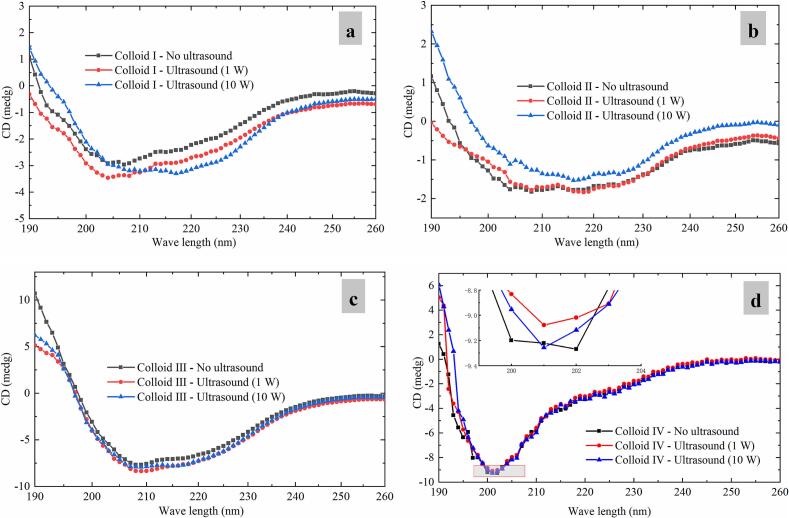
Far-UV CD spectra of casein-dextran colloids (Colloid Ⅰ (a), Colloid Ⅱ (b), Colloid Ⅲ (c) and Colloid Ⅳ (d)) with different ultrasonic powers.

Fig. 8 shows a board negative band ranging from 200 to 230 nm, which is characteristic of α-helix secondary structure [44]. Obviously, CZ-US applications increased the negative peak around 215 nm for Colloid Ⅰ (Fig. 8a) and decreased the negative peak around 215 nm for Colloid Ⅱ (Fig. 8b), of which all changes were less than 1 medg, indicating that the α-helix content changed slightly. In addition, under 10 W ultrasound treatment, an obvious red shift was observed for Colloid Ⅰ (Fig. 8a), suggesting a transition of casein conformation from disorder to order [45]. Meanwhile, Table 2 summarizes the secondary structure compositions of all samples, which shows that the impact of CZ-US on secondary structures of different casein-dextran colloids is almost negligible.

**Table 2:** Secondary structure compositions of the colloids with different ultrasonic powers.

Type	ultrasonic power (W)	α-helixes (%)	β-sheets (%)	β-turns (%）	Random coils (%)
Colloid Ⅰ	0	5.6 ± 2.1 ^a^	40.9 ± 3.8 ^a^	22.1 ± 0.6 ^a^	31.5 ± 1.1 ^a^
	1	5.7 ± 2.1 ^a^	40.9 ± 3.8 ^a^	22.1 ± 0.6 ^a^	31.5 ± 1.1 ^a^
	10	5.6 ± 2.1 ^a^	41.0 ± 4.1 ^a^	22.1 ± 0.6 ^a^	31.5 ± 1.1 ^a^
Colloid Ⅱ	1	5.5 ± 2.1 ^a^	40.8 ± 3.7 ^a^	23.0 ± 1.0 ^a^	30.0 ± 1.1 ^a^
	10	5.4 ± 2.3 ^a^	40.7 ± 3.8 ^a^	23.0 ± 1.0 ^a^	30.0 ± 1.1 ^a^
	0	6.6 ± 2.2 ^a^	40.5 ± 4.2 ^a^	23.0 ± 1.0 ^a^	30.0 ± 1.1 ^a^
Colloid Ⅲ	1	6.7 ± 2.2 ^a^	40.4 ± 4.0 ^a^	23.0 ± 1.0 ^a^	30.0 ± 1.1 ^a^
	10	6.6 ± 2.3 ^a^	40.4 ± 4.2 ^a^	23.0 ± 1.0 ^a^	30.0 ± 1.1 ^a^
	1	5.5 ± 2.1 ^a^	40.8 ± 3.7 ^a^	23.0 ± 1.0 ^a^	30.0 ± 1.1 ^a^
Colloid Ⅳ	0	5.9 ± 2.3 ^a^	41.3 ± 4.4 ^a^	22.5 ± 1.0 ^a^	30.3 ± 1.3 ^a^
	1	5.9 ± 2.3 ^a^	41.3 ± 4.4 ^a^	22.5 ± 0.9 ^a^	30.4 ± 1.3 ^a^
	10	5.9 ± 2.2 ^a^	41.2 ± 4.4 ^a^	22.4 ± 0.9 ^a^	30.6 ± 1.5 ^a^

Different lower cases letter in the same column shows significant differences (p < 0.05).

#### 3.3.4 Fourier transform infrared spectroscopy

Different functional groups of protein-polysaccharide colloids show different vibration and rotational frequencies in infrared spectroscopy, which can be used to identify different functional group compositions in the colloids [41].

**Fig. 9:**
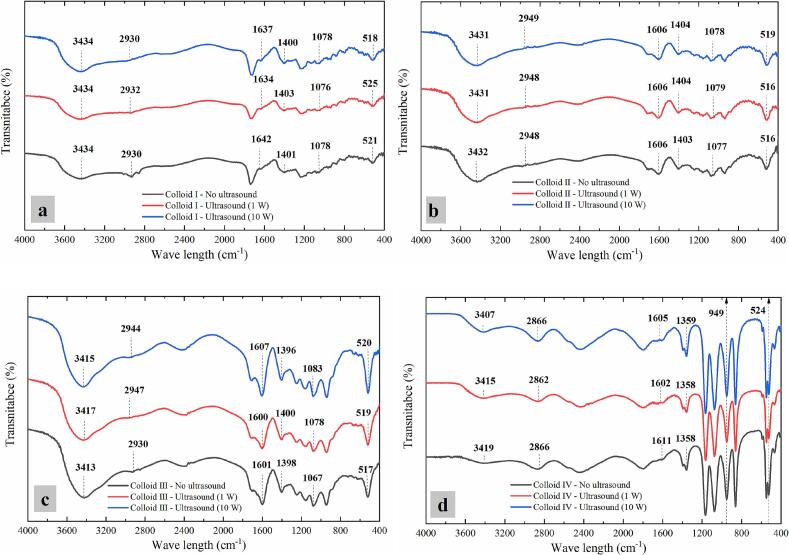
ATR-FTIR spectra of colloid mixture systems (Colloid Ⅰ (a), Colloid Ⅱ (b), Colloid Ⅲ (c) and Colloid Ⅳ (d)) treated by different ultrasonic powers.

Spectrum of different casein-dextran colloids showed the expected characteristic chemical groups (Fig. 9): broad and strong absorption bands at 3400 – 3435 cm^−1^ related to O—H stretching vibration, meanwhile the C—H stretching vibration and C=O stretching vibration (amide A) is indicated at 2860 – 2950 cm^−1^ and 1600 − 1665 cm^−1^ (Xiong et al., 2019b). Comparative analysis revealed distinct spectral characteristics among the colloidal systems. Colloid IV (pH 7.8) demonstrated a significant blue shift relative to Colloid I (pH 2.8) under non-ultrasonic conditions (Fig. 9a and d). Similarly, Colloid III (casein: dextran = 1:4) exhibited a pronounced blue shift compared to Colloid II (casein: dextran = 4:1) without ultrasound treatment (Fig. 9c and b). These observations indicated that the structural conformations of casein-dextran colloids were influenced by both pH conditions and casein-to-dextran ratios. Furthermore, compared with no ultrasound treated samples, there were no significant new spectral peak observed in the CZ-US treated casein-dextran colloid (shown in Fig. 9). This suggests that the energy generated through stable cavitation of microbubbles [25]during CZ-US application was insufficient to induce breakage or recombination of functional groups in the protein-polysaccharide colloidal structures.

## 4 Discussion

### 4.1 How does CZ-US effect the aggregation properties of different Colloids？

Many studies [15,23,27]pointed out that the particles were broken down by HI-US treatment. CZ-US treatments may not act with enough energy to break up large casein-dextran network aggregates. For the investigated four colloids of different structures, effects of CZ-US treatments on their aggregation properties are mainly related to their hydrophilic/hydrophobic amino acid groups exposure.

Clearly, as shown in Fig. 2 and Fig. 7, casein structures in four colloids were unfolded to expose buried hydrophilic/hydrophobic amino acid groups induced by 10 W CZ-US treatments. However, after CZ-US treatments, there were different exposure results for four original structures of colloids. At the isoelectric point of casein, dextran molecules exhibited surface adhesion to self-aggregating casein structures, with CZ-US treatment inducing increased exposure of hydrophobic amino acid groups in Colloids II and III. In contrast, at pH values deviating from the isoelectric point, dextran demonstrated distinct behavior: either penetrating and adhering within casein structures or undergoing self-crosslinking. Under these conditions, CZ-US treatment of Colloid I (pH 2.8) and Colloid IV (pH 7.8) resulted in enhanced exposure of polar hydrophilic amino acid groups in casein and increased accessibility of hydrophilic dextran moieties in the colloidal systems.

In addition, exposure of hydrophobic/hydrophilic amino acid groups also affected turbidity of these four casein-dextran colloids. Linke et al (2016) illustrated that the factors affecting turbidity contain particle size, insoluble matter content, refractive index and the composition of the aqueous phase. Therefore, particle size and insoluble matter content become the most important factors affecting turbidity for casein-dextran colloids in this work. For Colloid Ⅱ and Ⅲ (pH 4.8, at pl), when applying CZ-US, increased insoluble matter concentrations may lead their turbidity to increase because of more exposed hydrophobic amino acid groups of casein (shown in Fig. 4a). For Colloid Ⅰ and Ⅳ (pH was outside the casein isoelectric point), they showed lower insoluble matter concentrations with CZ-US treatments (shown in Fig. 2), and newly exposed hydrophilic amino acid groups of casein caused more casein and dextran to further aggregate and then form larger structures. Hence, Colloid Ⅰ and Ⅳ showed increased turbidity under CZ-US effects (shown in Fig. 4a).

### 4.2 How does CZ-US effect the structures and rheological behaviors of different Colloids？

In general, size distribution, particle size, particle deformability, and shape of a particle are the causes which effects the flow behavior of biopolymers [36,46,47]. Meanwhile, many researches [37,48] demonstrated that applying HI-US decreased the apparent viscosity of biopolymers because of the particle size reduction and network breaking. However, energy emerged by CZ-US treatment is limit, and it can barely break the particle network. Nevertheless, CZ-US treatment can unfold protein structures, enable the exposure of hydrophilic/hydrophobic amino acid groups, and then change biopolymers’ structures, thus affecting the rheological properties of protein-polysaccharide colloids.

Effects of CZ-US treatments on rheological behaviors of these colloids varied with different structural changes. When pH was at the casein isoelectric point (Colloid Ⅱ and Ⅲ), there were more hydrophobic amino acid groups of casein exposed with CZ-US treatments. As for Colloid Ⅱ (network, casein: dextran = 1: 4), under CZ-US, the binding patches on the casein particle interface were reduced for the dextran due to the exposure of more hydrophobic amino acid groups, therefore, more exceeded dextran was self-crosslinked leading to a denser gel network with higher yield stresses (Fig. 5b and Fig. 6b). However, in the case of Colloid Ⅲ (shear-thinning fluid, casein: dextran = 4: 1), the reduced binding patches under CZ-US then further excluded dextran from the casein-dextran colloid thus weakened the fluid consistency (Fig. 5c and Fig. 6c).

Otherwise, when pH was at other mediums rather than the casein isoelectric point (Colloid I and Ⅳ), there were more exposed hydrophilic amino acid groups of casein under CZ-US treatments. Hence, for Colloid Ⅰ (network, pH 2.8), more dextran adhered to the newly available binding patches of casein particle interface, thus the consistency of the casein-dextran network was disrupted, resulting in the decrease of yield stress under CZ-US (Fig. 5a). As for Colloid Ⅳ (Newtonian fluid, pH 7.8), more free dextran adhered into the casein interface thanks to more available binding sites under CZ-US, leading to the formation of larger aggregates/complex, exhibiting higher fluid consistency (shown in Fig. 5d, Azarikia and Abbasi, 2016).

### 4.3 Similarities and differences between CZ-US and HI-US

Above all, CZ-US treatments can unfold protein structures and then expose hydrophilic/hydrophobic amino acid groups to influence on aggregation, structural and rheological properties of protein-polysaccharide colloids. These effects have certain similarities and differences compared with HI-US.

#### 4.3.1 Similarities

Firstly, there is an obvious transformation of particle size distribution from multimodal to unimodal under CZ-US treatments with increasing ultrasonic powers (shown in Fig. 3a and Fig. 3b), and HI-US treatments are also reported to enable homogenize the size distribution of protein-polysaccharide colloids [26,27]. Secondly, protein structures were unfolded under CZ-US, which is consistent with HI-US treatments [18,49]. Finally, same effect as HI-US treatments [15,27], the functional groups of casein-dextran colloids remained unchanged after CZ-US treatments with limit energy.

#### 4.3.2 Differences

HI-US can bring about extreme effects including continuous heat, high pressure and high shear rates for biopolymers [37,50]. Obviously, the effects of CZ-US are much more modest in different aspects.

As the exposure of more hydrophilic/hydrophobic amino acid groups, the turbidities may increase (Fig. 4b) in protein-polysaccharide colloids under CZ-US treatments. However, many studies [15,27] on HI-US have found otherwise. Specially, the way that HI-US and CZ-US impact the rheological behaviors of colloids is quite different. Many researches demonstrated that HI-US lowered the apparent viscosity of colloids through breaking their structures (Kaltsa et al., 2013b; [48]or particle aggregating in high-temperature [51]. However, CZ-US affects the rheological behaviors of protein-polysaccharide colloids mainly achieved by unfolding certain protein structures, exposing hydrophilic and hydrophobic amino acid groups, and then altering the colloidal structures. Furthermore, HI-US treatment can change the amount of surface charge of protein and polysaccharide to form the more stable complex systems due to stronger electrostatic interaction [19], but CZ-US treatments had no significant influence on surface charge of casein-dextran colloids (Fig. 4a). Notably, the effect of CZ-US on colloidal microstructure, including conformation and protein secondary structure, is small or even negligible, apparently contrary to the findings of a large number of HI-US studies [19,20,23].

## 5 Conclusions

**Fig. 10:**
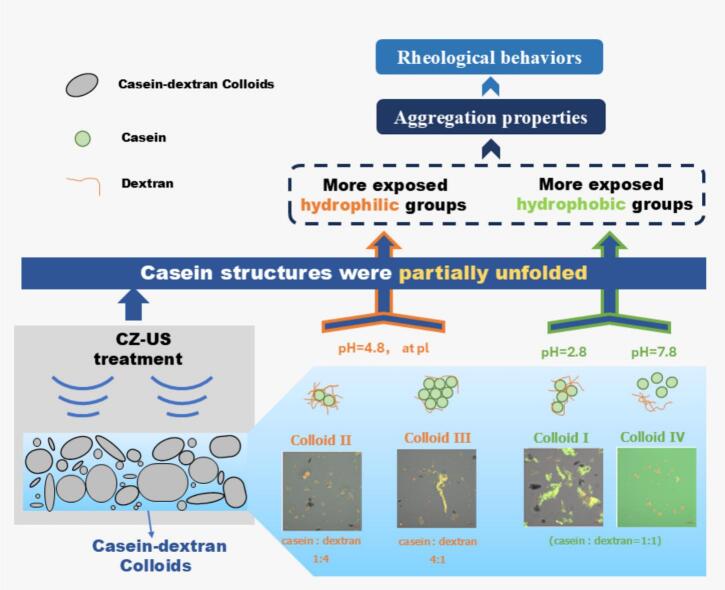
Diagram of the main effects of CZ-US treatment on the structures of various representative casein-dextran colloids.

CZ-US effects on the aggregation, rheological and structure properties of casein-dextran colloids were somewhat different from HI-US effects. In general, CZ-US unfolded the protein structure to expose hydrophobic/hydrophilic amino acid groups (shown in Fig. 10), and mildly affected the aggregation and flow behaviors, without altering the secondary structures of proteins and functional groups of casein-dextran colloids.

CZ-US enabled particle size distribution of casein-dextran colloids transform from multimodal to unimodal. However, the turbidity of casein-dextran colloids increased with the changes of aggregation and structure properties induced for colloids by CZ-US treatments. In particular, CZ-US induced a higher yield stress of Colloid Ⅱ (pH 4.8, casein: dextran = 1:4) and a greater fluid consistency of Colloid Ⅳ (pH 7.8, casein: dextran = 1:1) in this work.

CZ-US strongly depended on the solution medium of the colloids. As shown in Fig. 10, when pH was at the casein isoelectric point, more hydrophobic amino acid groups of casein were exposed under CZ-US treatments, the binding patches on the casein particle interface were therefore reduced for the dextran. When pH was at other mediums rather than the casein isoelectric point, more hydrophilic groups were exposed under CZ-US treatments, more dextran adhered into the casein interface thanks to more available binding sites. In conclusion, the effect of CZ-US on the properties of casein-dextran colloid was mild, that is, CZ-US-membrane separation coupling may offer better performance in maintaining the inherent characteristics of colloidal feed than HI-US-membrane separation coupling.

## CRediT authorship contribution statement

**Binsha Peng:** Writing – original draft, Investigation, Formal analysis. **Songlin Wen:** Investigation, Formal analysis. **Wenchong He:** Writing – review & editing. **Chongde Wu:** Writing – review & editing, Resources. **Jun Huang:** Resources, Methodology. **Rongqing Zhou:** Writing – review & editing, Resources. **Nicolas Hengl:** Writing – review & editing, Resources. **Frederic Pignon:** Writing – review & editing, Resources. **Yao Jin:** Writing – review & editing, Supervision, Funding acquisition, Conceptualization.

## Declaration of competing interest

The authors declare that they have no known competing financial interests or personal relationships that could have appeared to influence the work reported in this paper.

## Data Availability

The authors are unable or have chosen not to specify which data has been used.
